# Sequence Effect of Self-Assembling Peptides on the Complexation and *In Vitro* Delivery of the Hydrophobic Anticancer Drug Ellipticine

**DOI:** 10.1371/journal.pone.0001956

**Published:** 2008-04-09

**Authors:** Shan Yu Fung, Hong Yang, P. Chen

**Affiliations:** Department of Chemical Engineering, University of Waterloo, Waterloo, Ontario, Canada; Massachusetts Institute of Technology, United States of America

## Abstract

A special class of self-assembling peptides has been found to be capable of stabilizing the hydrophobic anticancer agent ellipticine in aqueous solution. Here we study the effect of peptide sequence on the complex formation and its anticancer activity *in vitro*. Three peptides, EAK16-II, EAK16-IV and EFK16-II, were selected to have either a different charge distribution (EAK16-II vs. EAK16-IV) or a varying hydrophobicity (EAK16-II vs. EFK16-II). Results on their complexation with ellipticine revealed that EAK16-II and EAK16-IV were able to stabilize protonated ellipticine or ellipticine microcrystals depending on the peptide concentration; EFK16-II could stabilize neutral ellipticine molecules and ellipticine microcrystals. These different molecular states of ellipticine were expected to affect ellipticine delivery. The anticancer activity of these complexes was tested against two cancer cell lines: A549 and MCF-7, and related to the cell viability. The viability results showed that the complexes with protonated ellipticine were effective in eradicating both cancer cells (viability <0.05), but their dilutions in water were not stable, leading to a fast decrease in their toxicity. In contrast, the complexes formulated with EFK16-II were relatively stable upon dilution, but their original toxicity was relatively low compared to that with protonated ellipticine. Overall, the charge distribution of the peptides seemed not to affect the complex formation and its therapeutic efficacy *in vitro*; however, the increase in hydrophobicity of the peptides significantly altered the state of stabilized ellipticine and increased the stability of the complexes. This work provides essential information for peptide sequence design in the development of self-assembling peptide-based delivery of hydrophobic anticancer drugs.

## Introduction

Self-assembling peptides are emerging nano-biomaterials with promising biomedical and bioengineering applications [1]–[3]. Among them is a special class of ionic-complementary peptides discovered from a yeast Z-DNA binding protein [4]. These peptides have a unique amphiphilic structure resulting from an alternative arrangement of hydrophobic and hydrophilic amino acids in sequence. They also consist of alternating positive and negative charges at physiological conditions, resulting in ionic complementarity. These peptides are capable of self-assembling into very stable nanostructures or macroscopic membranes, which can withstand high temperature, extreme pH, many digesting enzymes and denaturation agents [4], [5]. Moreover, they exhibit good biocompatibility with many cultured mammalian cells [6] and no detectable immune responses can be observed when being introduced into animals [4], [7], [8]. These properties make them ideal materials for tissue scaffolding [9]–[11], regenerative medicine [7], [8], [12] and drug delivery [13]–[17].

The ionic-complementary self-assembling peptides have recently been used as novel nano-biomaterials in the local delivery of hydrophilic peptide/protein drugs [16]–[19] and the formulation of hydrophobic chemotherapeutics [20], [21]. The biotinylated, self-assembling peptide RADA16-II was found to be able to locally deliver insulin-like growth factor 1 (IGF-1) to the myocardium, and the peptide nanofibers provided sustained release of IGF-1 for 28 days [16]. These peptide nanofibers could also bind with a human platelet derived growth factor (PDGF-BB) and deliver it *in vivo* with sustained release to successfully decrease cardiomyocyte death and preserve systolic function [17]. In addition to the delivery of peptide/protein drugs, it has recently been demonstrated that a self-assembling peptide, EAK16-II, can stabilize hydrophobic compounds in aqueous solution and release them into a cell membrane mimic in a control manner [14], [20]. Further studies revealed that such a peptide is capable of stabilizing the hydrophobic anticancer agent ellipticine with different molecular states in aqueous solution depending on the peptide and ellipticine concentration, which in turn affects the ellipticine release from the complexes [21]. These studies have shown great potential for the use of the self-assembling peptides in drug delivery.

However, current studies of using self-assembling peptides for drug delivery are still at their early stage. The development of a self-assembling peptide-based delivery system requires better design of peptide sequences for specific delivery goals. Previous studies have shown that a difference in the charge distribution of the self-assembling peptides significantly alters the nanostructure of the peptide assemblies [22], [23]. In addition, the charge distribution affects the peptide assemblies in response to solution pH [22]. The variations in peptide length, hydrophobicity and ionic complementarity have been applied to control the formation of self-assembling peptide matrices [24]. The resulting structure of peptide assemblies will impact the construction of delivery vehicles for different therapeutics. For example, delivery of protein or siRNA drugs requires the cell penetration ability while cell recognition is critical to achieve targeted delivery of anticancer therapeutics [25]–[27]. Therefore, proper design of peptide sequences becomes crucial to build functional peptide-based carriers for effective drug delivery.

In this work, we carry out the study of peptide sequence effects on the drug formulation and *in vitro* delivery. Three self-assembling peptides, EAK16-II, EAK16-IV and EFK16-II, are chosen to investigate the effects of charge distribution (type II vs. type IV) and hydrophobicity (alanine A vs. phenylalanine F). A hydrophobic anticancer agent, ellipticine, is selected as a model drug, following our early studies of this drug. The self-assembled nanostructures of these peptides are first characterized by atomic force microscopy (AFM); the hydrophobicity of the peptides dissolved in aqueous solution is studied via surface tension measurements, and fluorescence spectroscopy using a hydrophobic fluorescent probe. These characteristics of the three peptides are expected to impact their complexation with ellipticine, in terms of ellipticine molecular states and the size of the resulting complexes. The anticancer activity of the formulation is tested *in vitro* against two cancer cell lines: non-small cell lung cancer cell A549 and breast cancer cell MCF-7. The stability of the complexes after serial dilutions in aqueous solution is further investigated. The information obtained in this study is aimed at providing appropriate design principles for selecting peptide sequences, to construct advanced functional peptide carriers for anticancer drug delivery.

## Results and Discussion

The self-assembling peptide EAK16-II has been found to be able to stabilize the hydrophobic anticancer agent ellipticine in aqueous solution [20], [21]; the ellipticine release kinetics from the complexes into a cell membrane mimic has also been determined [21]. Here, we report how the subtle differences in the peptide sequence affect the properties of the peptide assemblies, the formation of the peptide-ellipticine complexes, and the cellular toxicity of the complexes.

### Sequence effect on the peptide assemblies

**Figure 1:**
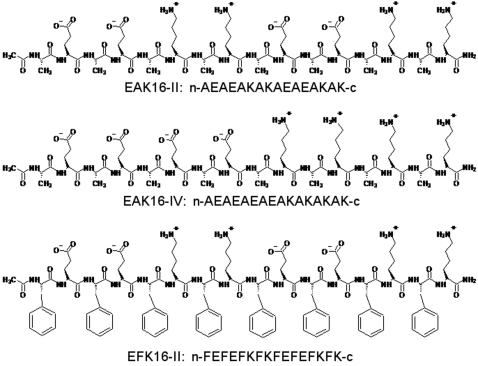
Molecular structures and sequences of EAK16-II, EAK16-IV and EFK16-II. N and C termini are protected by acetylation and amidation, respectively.

Three self-assembling, ionic-complementary peptides, EAK16-II, EAK16-IV and EFK16-II, are used in this study. The latter two peptides are derived from the first one EAK16-II. All peptides have 16 amino acids in sequence with 3 amino acid components: E, K and A or F, as shown in Figure 1. EAK16-IV has a different charge distribution of type IV (−−−−++++) from EAK16-II as type II (−−++−−++), while the difference between EFK16-II and EAK16-II is a more hydrophobic residue F replacing A in EAK16-II. The slight differences in sequence among the three peptides may significantly affect their assemblies and further complexation with the hydrophobic molecules.

**Figure 2:**
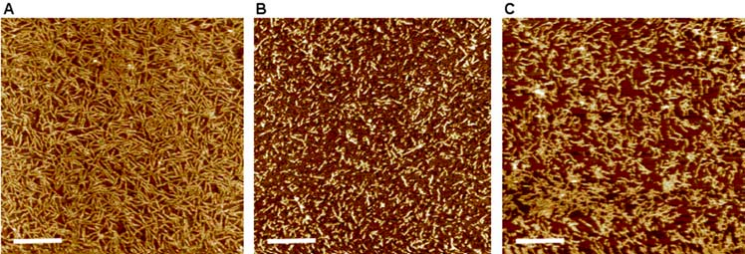
AFM images of the peptide nanostructures: (a) EAK16-II; (b) EAK16-IV; (c) EFK16-II. The peptide concentration is 0.5 mg/mL. The scale bar is 200 nm.

First, the peptide self-assembled nanostructures are found to be different among the three peptides. The distribution of negative and positive charges towards the two ends of an EAK16-IV molecule at neutral pH is reported to cause the folding of the peptide molecule to form a β-turn structure, resulting in the formation of globular nanostructures [22], [23]. EAK16-II, on the other hand, has a preferable stretched molecular structure and likely self-assembles into β-sheet rich nanofibers [23]. The nanostructures of the two peptides are shown in Figure 2a and b at a peptide concentration of 0.5 mg/mL. EAK16-II forms straight nanofibers, connecting to networks (Figure 2a), whereas EAK16-IV self-assembles into many more globular aggregates and some short nanofibers (Figure 2b). The formation of short nanofibers of EAK16-IV may be due to a relatively low pH (<5) at such a high peptide concentration: when the pH is low enough, some of the negatively charged residues can be neutralized so that the intramolecular ionic interaction is weakened. Thus, some peptides remain in a stretched form, facilitating the formation of nanofibers [22].

The nanostructures of EFK16-II are also different from those of EAK16-II as shown in Figure 2. EFK16-II forms predominant nanofibers and these fibers tend to aggregate into fiber clusters. This aggregation of nanofibers is probably due to a stronger hydrophobic interaction between them. Such a stronger hydrophobic interaction is expected to come from the more hydrophobic phenylalanine (F) residues in the EFK16-II sequence, compared with the alanine (A) residues in EAK16-II. This is probably why the nanofibers of EFK16-II tend to form fiber clusters, but those of EAK16-II are dispersed and form fiber networks.

**Figure 3:**
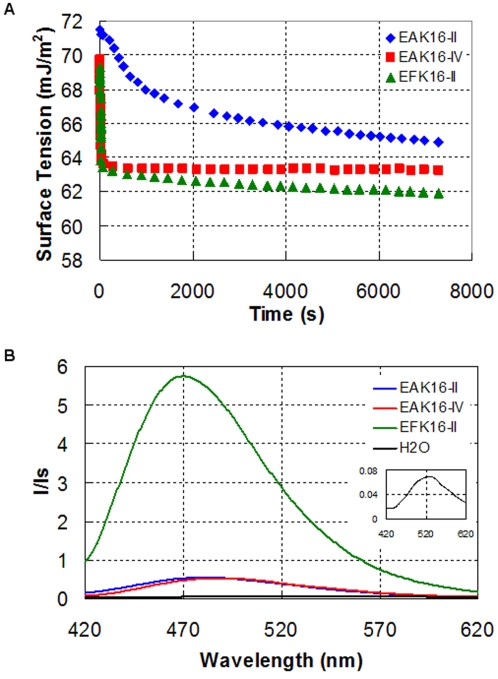
The hydrophobicity of the three peptides and their assemblies by dynamic surface tension (a) and ANS fluorescence (b). The inset is the ANS fluorescence control with the absence of peptides. The peptide concentration is 0.5 mg/mL, and the ANS concentration is 10 µM.

The hydrophobicity of the three peptides and their assemblies is further characterized by surface activity and fluorescence measurements, and the results are shown in Figure 3. Figure 3a shows the surface tension as a function of time for the three peptides at a peptide concentration of 0.5 mg/mL. For each profile, the surface tension decreases fast initially and slowly approaches equilibrium. This change with time corresponds to the dynamic process of the adsorption of peptide molecules/assemblies at the air-liquid interface, leading to the decrease in surface tension [28]. Comparing the surface tensions of the three profiles at 2 h (near equilibrium), they follow a trend: EAK16-II>EAK16-IV>EFK16-II. In general, the lower the surface tension is, the more hydrophobic the molecule is. Thus, the hydrophobicity of the three peptides and their assemblies (coexisting in solution) has a reversed trend: EFK16-II>EAK16-IV>EAK16-II. This is reasonable that EFK16-II is the most hydrophobic peptide among the three as it consists of phenylalanine residues, which is more hydrophobic than alanine residues in EAK16-II and EAK16-IV. The reason why EAK16-IV has a lower equilibrium surface tension than EAK16-II is probably due to the formation of β-turn structure through intramolecular ionic interaction in EAK16-IV. This conformational change may cause the exposure of hydrophobic alanine residues toward the aqueous phase, resulting in a slight increase in hydrophobicity of the molecule and lowering the surface tension [22].

Figure 3b shows the fluorescence spectra of the ANS probe in the three peptide solutions comparing to that in pure water (black line and the inset). The normalized fluorescence intensities of ANS in different solutions follow a trend: EFK16-II>>EAK16-II≈EAK16-IV>H_2_O. Meanwhile, the peak positions of the spectra are different; it locates at ∼520 nm in pure water (inset), but shifts to ∼485 nm in EAK16-II and EAK16-IV solutions. The ANS fluorescence spectrum has a peak of ∼470 nm in the EFK16-II solution. The changes in ANS fluorescence intensity and peak position indicate that the ANS probe is in different environments. ANS is a widely used probe to study protein aggregation as well as cell membrane composition and function due to its extreme sensitivity to the changes in the polarity of the probed environment [29]–[31]. A less polar environment will cause a shift of the fluorescence spectrum of ANS toward lower wavelengths (blue shift) and a significant increase in the fluorescence quantum yield [31]. Thus, the changes in ANS fluorescence in different peptide solutions (Figure 3b) can be related to the hydrophobicity of the local environment where ANS resides. This leads to a conclusion that EFK16-II provides a more hydrophobic environment for ANS than the other two peptides. These results also indicate that EFK16-II may have different impacts on the complexation with the hydrophobic anticancer agent ellipticine, compared with EAK16-II and EAK16-IV.

It is worth noting that the hydrophobicity determined by the two methods may refer to two different situations. Surface tension is a solution property and based on the molecular adsorption at the interface, affecting the surface free energy. The adsorption process involves three steps: i) diffusion of the molecules from the bulk to the sub-interface; ii) transfer of the molecules from the sub-interface to the interface; iii) rearrangement of the molecules at the interface [32]. Considering diffusion to be the rate limiting step, small molecules are expected to rapidly accumulate at the interface due to their faster diffusion rate than large ones. Thus, in the self-assembling peptide systems, the surface tension may reflect predominantly the properties of peptide monomers and small peptide assemblies, rather than those of the large peptide aggregates. On the other hand, ANS fluorescence depends pronouncedly on the local probe environment. The binding of ANS to peptide monomers may not significantly affect its fluorescence properties as it still “feels” surrounding solvent molecules (i.e., water in this case). Only when the ANS probe is enclosed in a different environment from the solvent does its fluorescence greatly change. Therefore, the observed changes in ANS fluorescence in Figure 3b should result from the properties of peptide assemblies/aggregates. This is probably why the difference between EAK16-II and EAK16-IV from surface tension is not observed by the ANS fluorescence.

### Sequence effect on the complex formation

**Figure 4:**
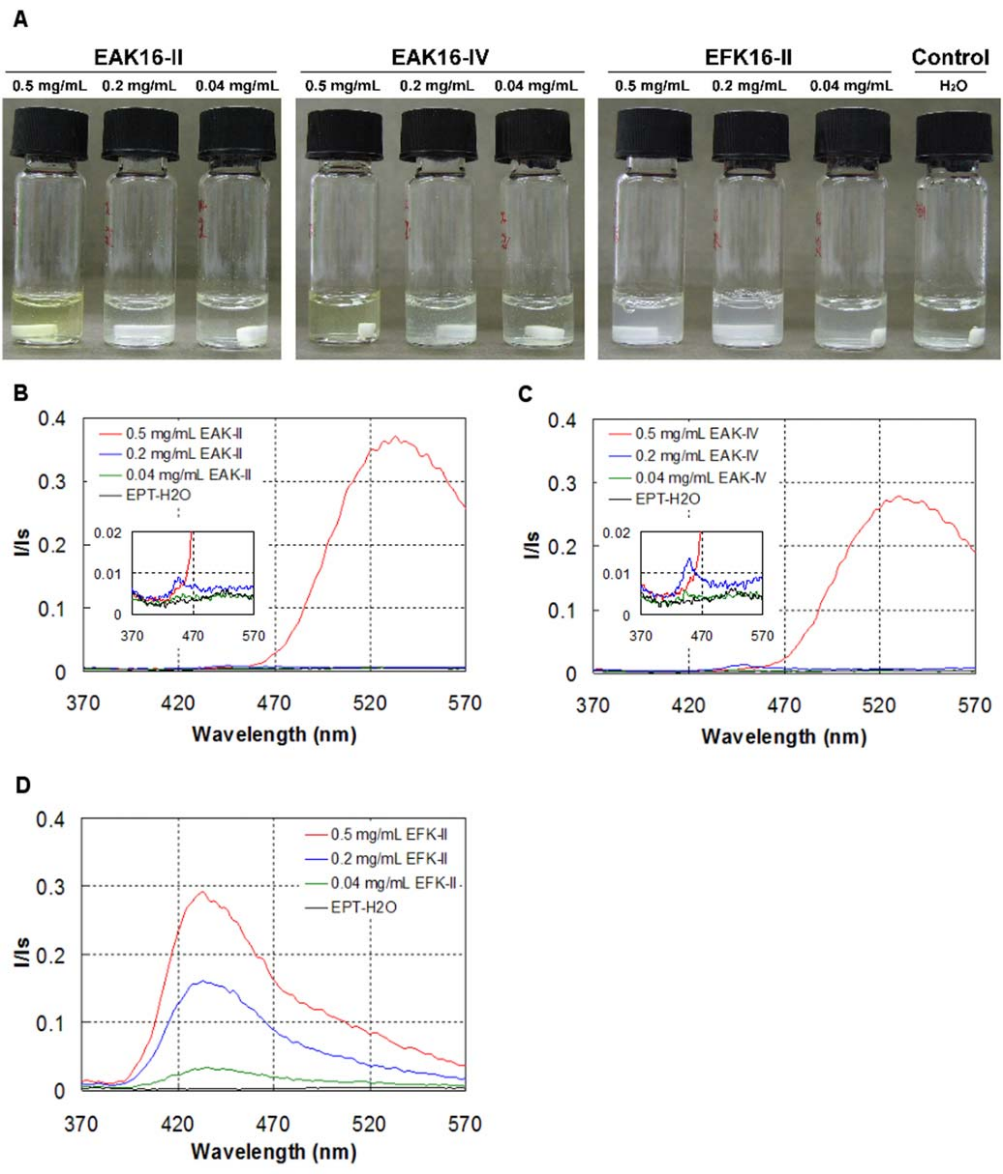
The formation of peptide-ellipticine complexes. (a) Photographs of the complexes with the three peptides at different peptide concentrations and the ellipticine in pure water as a control. The normalized fluorescence spectra of ellipticine in the complexes with EAK16-II (b), EAK16-IV (c) and EFK16-II (d). The insets show the spectra of the complexes with low peptide concentrations.

It is found above that different peptide sequences affect the peptide assemblies and their properties. Such effects further influence the formation of peptide-ellipticine complexes. The results are shown in Figure 4. The differences among the complexes made of the three peptides can be directly visualized from the appearance of the suspensions (Figure 4a). For EAK16-II, the peptide-ellipticine solutions appear to be slightly turbid at peptide concentrations of 0.2 and 0.04 mg/mL, indicating the formation of large colloidal suspensions. However, at a concentration of 0.5 mg/mL, the solution becomes clearer with a light yellow color (far left vial). Similar appearances of the peptide-ellipticine solutions are found for EAK16-IV (central three vials) except that the solution looks less yellow at a peptide concentration of 0.5 mg/mL. For EFK16-II, all solutions look cloudy. Compared with the control sample (with the absence of peptides, far right vial) that remains colorless and transparent, the changes in the solution appearance of the peptide-ellipticine samples reveal that ellipticine has been uptaken by the peptides and stabilized in solution.

The different appearances of the solutions may indicate different molecular states of ellipticine in the complexes. Recent studies on the complexation of EAK16-II with ellipticine have demonstrated that two molecular states of ellipticine, either protonated or crystalline, can be obtained in the complexes depending on the peptide and ellipticine concentrations [21]. The protonation of ellipticine usually occurs at a higher peptide concentration, related to a relatively low solution pH (<5, pKa of ellipticine is ∼6) [33]; protonated ellipticine can be stabilized by ionic interaction with the negatively charged residues (glutamic acid E in this case) of the peptide. The ellipticine microcrystals are stabilized by peptide assemblies coating on the surface [20], [21]. When ellipticine is protonated, it can dissolve in aqueous solution and cause the solution to have a yellow, transparent appearance. On the other hand, the suspended ellipticine microcrystals make the solution turbid and cloudy. Thus, by looking at the appearance of the samples, one can possibly predict that EAK16-II and EAK16-IV can stabilize protonated or crystalline ellipticine while ellipticine stabilized by EFK16-II may be predominantly in microcrystal form.

The molecular state of ellipticine can be further elucidated by the ellipticine fluorescence spectra. It has been found that protonated ellipticine molecules have a fluorescence peak at ∼520 nm while the fluorescence peak at ∼430 nm is attributed to neutral ellipticine molecules [34]; crystalline ellipticine exhibits a fluorescence peak at ∼470 nm with an extremely low intensity [20]. The fluorescence spectra of the complexes with the three peptides, EAK16-II, EAK16-IV and EFK16-II, are shown in Figure 4b, c and d, respectively. For EAK16-II and EAK16-IV, the complexes with 0.5 mg/mL peptide have a fluorescence peak located ∼520 nm, indicating that ellipticine is protonated. At peptide concentrations below 0.5 mg/mL, the spectra have a peak close to 470 nm with an extremely low intensity (insets in Figure 4b and c), representing crystalline ellipticine. Interestingly, the complexes with EFK16-II exhibit a fluorescence spectrum with a major peak located at ∼435 nm and a small shoulder covering the wavelengths from 470 to 570 nm (Figure 4d), very different from those of protonated and crystalline ellipticine. The peak located at ∼435 nm represents neutral (non-charged) ellipticine, present as individual molecules in a much less polar environment [34]. The peak intensity is proportional to the EFK16-II concentration. These results indicate that EFK16-II can stabilize neutral, molecular ellipticine in aqueous solution; in contrast, the other two molecular states of ellipticine, protonated and crystalline, can be formed in the complexes with EAK16-II and EAK16-IV. EFK16-II assemblies provide a more hydrophobic environment than those of EAK16-II and EAK16-IV as shown in Figure 3b, possibly facilitating the stabilization of neutral ellipticine molecules. Note that in addition to neutral ellipticine, crystalline and protonated ellipticine can coexist in the suspensions as indicated by the turbid appearance of the suspensions and a shoulder from the fluorescence spectra. The fluorescence signals from crystalline ellipticine, however, are too small to be seen compared to those of neutral ellipticine. The different quantum yields and overlapping of the fluorescence signals from the three molecular states of ellipticine make it difficult to determine the percentage of each state among the three in the complexes. However, the total amount of stabilized ellipticine can be obtained.

**Figure 5:**
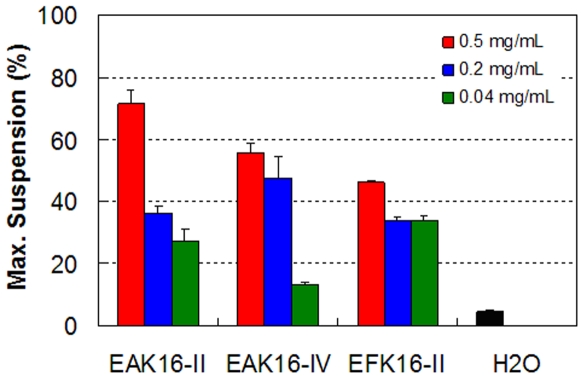
The maximum suspension (%) of ellipticine in aqueous solution stabilized by the three peptides and with the absence of peptides.

To determine how much ellipticine that can be stabilized in solution by the peptides, aliquots of the peptide-ellipticine suspensions were diluted into DMSO, and the UV absorption of ellipticine was collected. The ellipticine absorbance was then converted to corresponding ellipticine concentration in the suspensions. This concentration was compared with the given ellipticine concentration (0.04 mg/mL) to obtain the maximum suspension (%) as shown in Figure 5. Initially in the preparation, ellipticine is in solid form as a thin film at the bottom of the vial. With the help of the peptides and mechanical stirring over time, ellipticine can be uptaken and stabilized in the solution as protonated, neutral or crystalline ellipticine. Not all given ellipticine can be stabilized and suspended in solution; the deposition of ellipticine thin film can still be observed at the bottom of most sample vials. The amount of stabilized ellipticine varies with the types of peptides and peptide concentrations. The highest maximum suspension is found to be ∼71% (by wt.) by 0.5 mg/mL EAK16-II. At the same peptide concentration, such a value decreases to ∼56% for EAK16-IV and to ∼46% for EFK16-II. The lowest maximum suspension appears to be ∼13% by 0.04 mg/mL EAK16-IV, which is 3 folds higher than the control (∼4.5%) with the absence of peptides. The amount of ellipticine suspended by the peptide in water is found to be much higher than the reported solubility in water (∼0.6 µM) [35]. With the peptide concentration, the maximum suspension varies largely for EAK16-II and EAK16-IV but not for EFK16-II. Overall, EAK16-II appears to be the most effective peptide among the three at stabilizing protonated ellipticine (at a high peptide concentration of 0.5 mg/mL); EFK16-II, on the other hand, can stabilize neutral ellipticine (in addition to crystalline and protonated ellipticine), and it has less variation in the maximum suspension with different peptide concentrations.

### Size of the complexes

**Figure 6:**
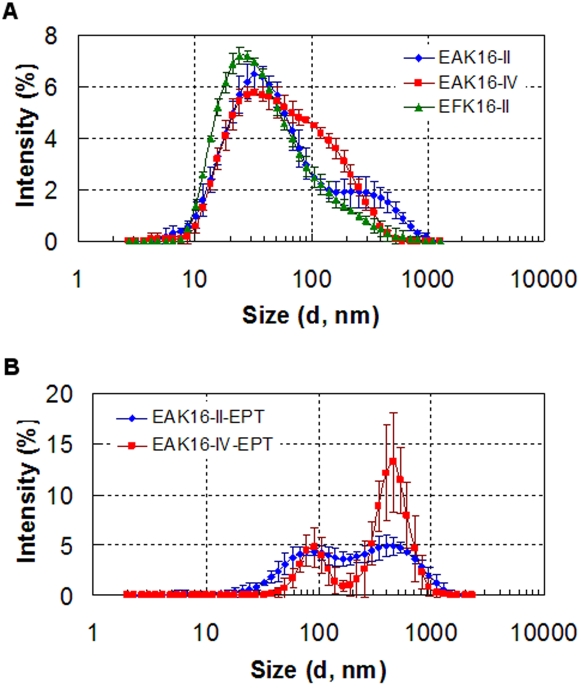
The size distribution of the three peptides at 0.5 mg/mL in pure water (a) and the complexes with 0.5 mg/mL EAK16-II and EAK16-IV (b) by DLS. EPT: ellipticine.

The size distribution of the peptide assemblies and complexes at a peptide concentration of 0.5 mg/mL is shown in Figure 6. For all three peptides, the peptide assemblies have a broad size distribution from 10 to several hundred nanometers (Figure 6a). They all have a major size population around 30 nm and a second one corresponding to a shoulder located at ∼300 nm, 100 nm and 200 nm for EAK16-II, EAK16-IV and EFK16-II, respectively. The size distribution of EAK16-II obtained here correlates well with our earlier findings, and the two populations represent short peptide nanofibers and fiber clusters [36]. When the peptides interact with ellipticine to form complexes, the size distributions change significantly as shown in Figure 6b. Note that only the size distributions of the complexes with EAK16-II and EAK16-IV are shown in the plot because the size of the complexes with EFK16-II is very polydispersed and over the detection limit of the instrument. The EAK16-II-ellipticine complexes have a relatively wider size distribution than EAK16-IV-ellipticine complexes; two size populations with one around 90 nm and the other around 500 nm can be found in both distributions.

**Figure 7:**
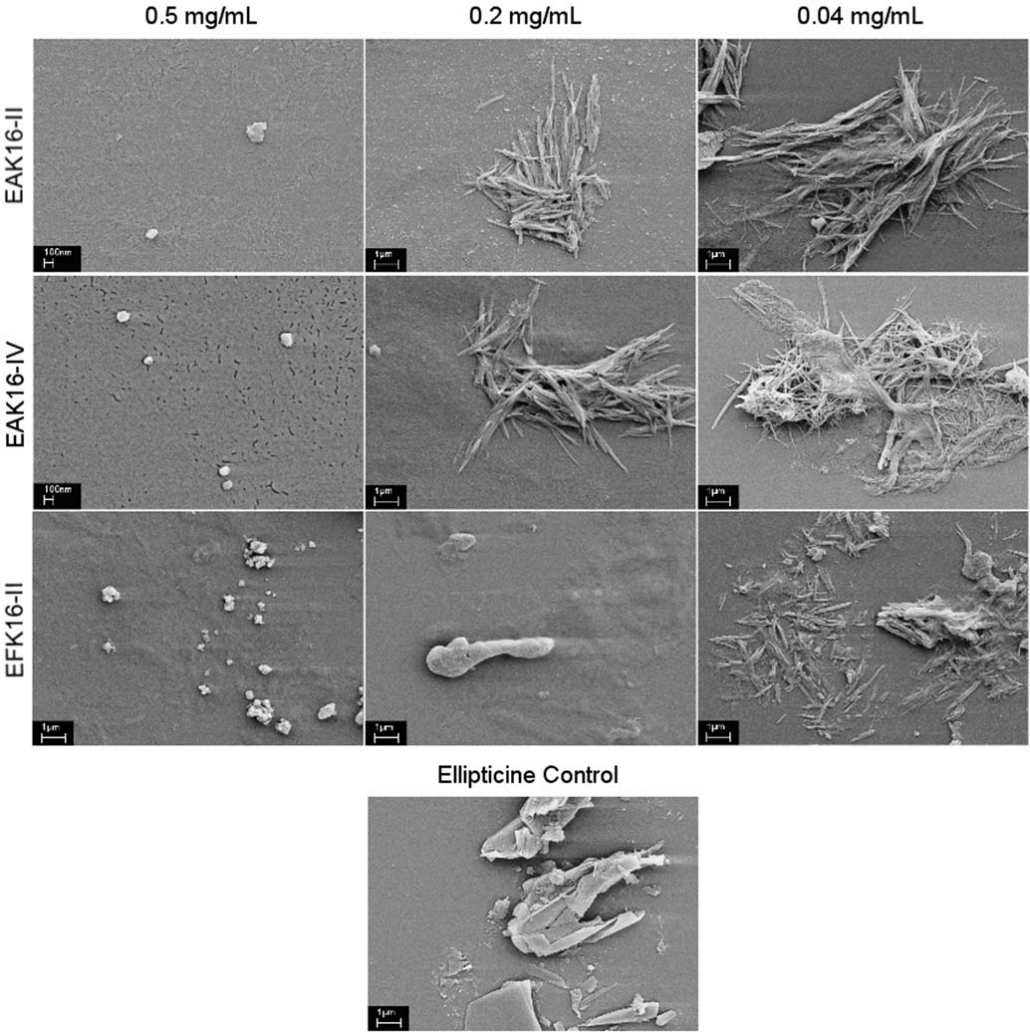
SEM images of the complexes with the three peptides at different peptide concentrations and ellipticine crystals in pure water as the control.

SEM imaging was applied as a complementary method to examine the size and morphology of the complexes for the three peptides at different peptide concentrations. The representative images are shown in Figure 7. It is clearly seen that the dimensions of the complexes with 0.5 mg/mL EAK16-II and EAK16-IV are in the range of ∼100–200 nm. For these two peptides, at peptide concentrations below 0.5 mg/mL, the size of the complexes can be as large as several micrometers. These complexes tend to have a rod-like or fiber-like structure, aggregating into bundles or entanglements. Such structures are very different from ellipticine crystals suspended in water (control).

For EFK16-II, the dimensions of the complexes range from hundreds of nanometers to several micrometers regardless of the peptide concentrations. However, the morphology of these complexes looks different according to the peptide concentration. At 0.04 mg/mL, the majority of the complexes are also rod-like although they seem to be shorter and more dispersed than those with EAK16-II and EAK16-IV; at higher peptide concentrations, the complexes appear to have irregular shapes. In addition, more membrane-like structures are observed in the background with the increase in EFK16-II concentration. These membrane-like EFK16-II assemblies could play an important role in stabilizing neutral ellipticine molecules. This may explain the increase in the fluorescence intensity of neutral ellipticine as a function of EFK16-II concentration shown in Figure 4d. Meanwhile, the ellipticine microcrystals could be stabilized by the amphiphilic EFK16-II monomers and small assemblies via forming peptide coatings on the surface of the crystals, leading to the formation of cloudy suspensions at all peptide concentrations.

### Cellular toxicity of the complexes and their dilutions

From the characterization of the complexes above, it can be summarized that peptide sequence will affect the molecular state of ellipticine in the peptide-ellipticine complexes/assemblies. EAK16-II and EAK16-IV can solubilize protonated ellipticine or encapsulate ellipticine microcrystals, depending on the peptide concentration. EFK16-II, on the other hand, can stabilize neutral ellipticine molecules in addition to the other two states in aqueous solution; the amount of neutral ellipticine that can be carried by EFK16-II assemblies is peptide concentration dependent. The size and structure of the complexes also depend on the type of peptide and peptide concentration. To gain more insight concerning these differences in the molecular state of ellipticine as well as the size and structure of the complexes, we investigated their cellular toxicity against two cancer cell lines and the stability of the complexes upon dilution in water. The information regarding the complex stability after dilution would be useful for later animal studies and preclinical experiments.

**Figure 8:**
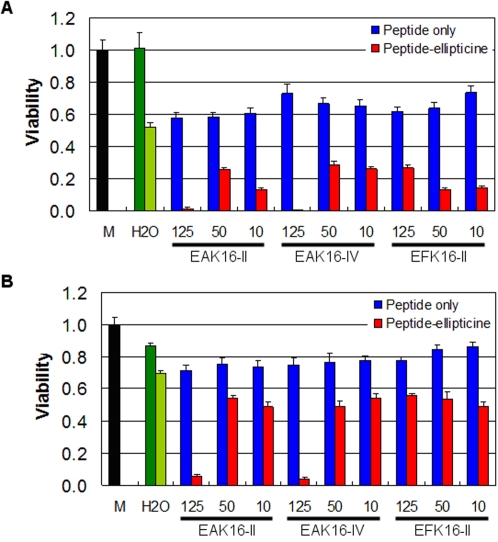
Cellular toxicity of the peptides and their complexes with ellipticine for A549 cells (a) and MCF-7 cells (b). The viability of non-treated cells is 1 (M: cells were treated with culture medium). For the solvent control, cells were treated with pure water (dark green bar); for the drug control, cells were treated with ellipticine in pure water with the absence of peptides (light green bar). Blue bars represent the peptide controls where no ellipticine was added.

Figure 8 shows the viability of both A549 and MCF-7 cancer cells upon being treated with peptide-ellipticine complexes for 48 h. For A549 cells (Figure 8a), all peptide-ellipticine complexes reduce the cell viability to less than 0.3 compared with the viability of non-treated cells (viability is 1). The toxicity of complexes is 2-folds higher than that of the ellipticine control with the absence of peptides (light green bar). The peptide controls have some toxicity to the cells, causing the decrease of viability to the values between 0.6 and 0.8. The much lower cell viability resulted from the peptide-ellipticine complexes compared with that from the ellipticine control is probably due to the fact that the peptides can stabilize large amounts of ellipticine in aqueous solution as shown in Figure 5. Interestingly, the cells treated with the complexes formulated with 0.5 mg/mL EAK16-II and EAK16-IV have almost zero viability. This may indicate that protonated ellipticine is more effective at killing A549 cells than other forms of ellipticine in the complexes. Such a result seems to contradict to the already known fact that neutral ellipticine is the active form to suppress the cancer cell growth [33].

The high efficacy of protonated ellipticine against cancer cells may be explained in the following. First, the protonated ellipticine has a positive charge, which can interact with a negatively charged cell membrane surface, leading to accumulation of ellipticine at the cell membrane surface. In addition, such a small molecule with a hydrophobic characteristic is expected to cross the cell membrane easily into the cytoplasm. Second, the protonated ellipticine molecules release much faster from the complexes compared with that from ellipticine microcrystals, due to the differences in complex size and a relatively weak interaction between protonated ellipticine and the peptide in the complexes [21]. This accelerates the diffusion speed of ellipticine from the complexes to the cells, facilitating a fast cellular uptake of ellipticine. Third, although EFK16-II is capable of stabilizing neutral ellipticine molecules, the amount of stabilized molecules are probably low; the release rate can be slow due to a possibly stronger hydrophobic interaction between neutral ellipticine and EFK16-II in the complexes. This is probably why the complex prepared with 0.5 mg/mL EFK16-II has much less effect on the cellular toxicity than protonated ellipticine stabilized by EAK16-II and EAK16-IV at the same peptide concentration.

For MCF-7 cells, the efficacy of protonated ellipticine on anti-proliferation of the cells becomes more significant when compared with the other forms of ellipticine (Figure 8b). The lowest cell viability for the complexes with neutral ellipticine and/or ellipticine crystals is around 0.5, which is about 70% of the viability for the ellipticine control (∼0.7). This percentage can be as low as ∼25% in the case of A549 cells. Such a difference may imply that the peptide-ellipticine suspensions are less effective to MCF-7 cells than to A549 cells. However, the complexes with protonated ellipticine have similar efficacy at killing both cells, although the reason behind is still unclear. It could be related to the different sensitivity, internalization path way and/or cell defense mechanism of the two cells in response to ellipticine. Nevertheless, these results provide evidence that the molecular state of ellipticine in the complexes significantly affects their cellular toxicity. Accordingly, one should be aware that selection of an appropriate formulation method is important in treating different cancer cells.

**Figure 9:**
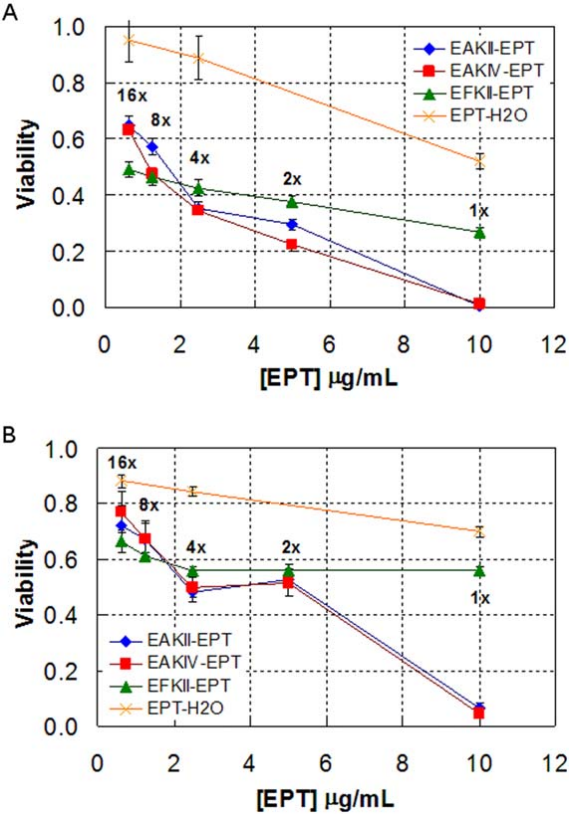
Cellular toxicity of the complexes formulated with the three peptides at a peptide concentration of 0.5 mg/mL and their serial dilutions in water for A549 cells (a) and MCF-7 cells (b). EPT: ellipticine.

Figure 9 shows the toxicity of the complexes with 0.5 mg/mL EAK16-II, EAK16-IV and EFK16-II upon serial dilution in water against both cell lines. The ellipticine control is diluted the same way for comparison. It is clearly seen that dilution has significant effect on the toxicity of the complexes with EAK16-II and EAK16-IV, where ellipticine is stabilized in protonated form. For A549 cells (Figure 9a), the cell viability is very low and less than 0.05 with these complexes before dilution; it increases largely to above 0.6 for 16 times dilution of the complexes. A similar trend is found for MCF-7 cells as the viability increases from less than 0.05 to above 0.7 (Figure 9b). Such changes imply that the complexes may not be stable, altering the protonated form of ellipticine after dilution in water. This instability of complexes is probably due to the rising of solution pH, leading to the deprotonation of ellipticine and the formation of ellipticine microcrystals after dilution. This may explain why a sudden increase in cell viability occurs upon 2 times dilution for MCF-7 cells as they seem to be more sensitive to protonated ellipticine than ellipticine microcrystals.

The EFK16-II-ellipticine complexes, on the other hand, exhibit good stability upon dilution in water. The viability increases from ∼0.25 to ∼0.5 for A549 cells; for MCF-7 cells, it remains unchanged at ∼0.57 up to 4 times dilution and then slightly increases to ∼0.65 for 16 tines dilution. Such a good stability may result from a stronger interaction between EFK16-II and ellipticine in the complexes due to a higher hydrophobicity of the peptide. In addition, a possible increase in solution pH after dilution should not affect the state of the stabilized neutral ellipticine molecules or ellipticine microcrystals. It is worth noting that although these complexes are not as effective as protonated ellipticine at killing cancer cells, their stability is much better, which is especially important for practical applications in clinics where drug dilution always occurs after administration into the bloodstream.

Overall, this study has demonstrated the effect of peptide sequence on its ability at stabilizing hydrophobic ellipticine in protonated, neutral as well as crystalline forms in aqueous solution. The difference in charge distribution (type II vs. type IV) on the peptide sequence seems not to have much effect on the complex formation and the molecular state of ellipticine in the complexes. The size, anticancer activity and stability of the complexes are very similar, although the charge distribution does affect, to some degree, the peptide assemblies: nanofibers vs. globular aggregates. It may be because the complexation of ellipticine with EAK16-II and EAK16-IV is mainly based on the peptide monomers but not on the peptide assembles. The increase in hydrophobicity of the peptide by replacing alanine (A) with phenylalanine (F), however, significantly alters the molecular state of ellipticine in the complexes, the complex stability and its therapeutic effect due to the following reasons: (i) the EFK16-II assemblies provide a more hydrophobic, enclosed environment where neutral ellipticine molecules can be solubilized; (ii) a stronger hydrophobic interaction between ellipticine and EFK16-II may further enhance the stability of the complexes upon dilution.

Different peptide sequences have different advantages in formulating the ellipticine drug. For example, 0.5 mg/mL EAK16-II (or EAK16-IV) can solubilize protonated ellipticine in nanoscale complexes with high anticancer activity against both A549 and MCF-7 cells, but these complexes are pH sensitive and not very stable after dilution. In contrast, the complexes formulated with 0.5 mg/mL EFK16-II are more stable upon dilution, but most of their sizes are in the micrometer range and their anticancer activity is relatively low. Nevertheless, these results provide essential information to design an appropriate peptide sequence that would optimize the delivery of hydrophobic anticancer drugs. One could utilize the advantages of different molecular states of ellipticine to improve the delivery efficacy, through a proper peptide design to form a stable, peptide nanocarriers, which can encapsulate neutral or crystalline ellipticine; if such a carrier enters cells through endocytosis, the encapsulated ellipticine becomes protonated at low pH in the lysosomes, and the protonated ellipticine can be released and cross the lysosome membrane into cytoplasm.

In conclusion, three ionic-complementary self-assembling peptides, EAK16-II, EAK16-IV and EFK16-II, with different charge distributions and hydrophobicities were found to be able to stabilize the hydrophobic anticancer agent ellipticine in aqueous solution. Ellipticine was stabilized in the form of microcrystals, protonated and neutral molecules depending on the peptide sequence and the peptide concentration. 0.5 mg/mL EAK16-II and EAK16-IV stabilized protonated ellipticine to form nano-complexes while crystalline ellipticine was obtained in the complexes with these peptides at lower peptide concentrations. On the other hand, EFK16-II was able to stabilize both neutral and crystalline ellipticine within the range of tested peptide concentrations; the amount of neutral ellipticine that can be stabilized was proportional to the peptide concentration. The different molecular states of stabilized ellipticine in the complexes greatly affected the anticancer activity of the complexes and their stability upon dilution in water. The complexes with protonated ellipticine were found to be very effective at killing both A549 and MCF-7 cells with a cell viability close to zero; however, these complexes were not very stable and their anticancer activity reduced significantly after serial dilution in water. The complexes formulated with EFK16-II (containing neutral ellipticine and ellipticine microcrystals), on the contrary, appeared to be stable after serial dilution, although their original anticancer activity was relatively low. These results showed that the differences in charge distribution of the peptides did not have much effect on the complex formation and their cellular toxicity, whereas the increase in peptide hydrophobicity could strengthen the interaction between the peptide and ellipticine, which gives the stability of their complexes upon dilution. This study provides necessary information on peptide sequence design to construct functional peptide carriers for hydrophobic anticancer drug delivery.

## Materials and Methods

### Materials

Three self-assembling, ionic-complementary peptides EAK16-II (Mw = 1657 g/mol, crude), EAK16-IV (Mw = 1657 g/mol, crude) and EFK16-II (Mw = 2265 g/mol, crude) were obtained from CanPeptide Inc. (Pointe-Claire, Quebec, Canada) and used without further purification. The mass spectra and HPLC data are presented in the Supporting Information (Figures S1, S2, S3, S4, S5). Their sequences and molecular structures are shown in Figure 1, where A corresponds to alanine, F to phenylalanine, E to glutamic acid and K to lysine. The N-terminus and C-terminus of the peptide were protected by acetyl and amino groups, respectively. At pH∼7, A and F are neutral, while E and K are negatively and positively charged, respectively. The anticancer agent ellipticine (99.8% pure) and 1-anilinonaphthalene-8-sulfonic acid (ANS) were purchased from Sigma-Aldrich (Oakville, ON, Canada) and used as received. Tetrahydrofuran (THF, reagent grade 99%) and dimethyl sulfoxide (DMSO, spectral grade >99%) were from Calendon Laboratories Ltd. (Georgetown, ON, Canada) and Sigma-Aldrich (Oakville, ON, Canada), respectively. Cell culture reagents including Dulbecco's modified eagle medium (DMEM), fetal bovine serum (FBS) and trypsin-ETDA were purchased from Invitrogen Canada Inc. (Burlington, ON, Canada). Phosphate buffer saline (PBS) and penicillin-streptomycin (p/s, 10000 U) were obtained from MP Biomedicals Inc. (Solon, OH, USA).

### Sample preparation

Appropriate amounts of the peptide powder were first dissolved in pure water (18 MΩ; Millipore Milli-Q system) to obtain fresh peptide solutions at concentrations of 0.5, 0.2 and 0.04 mg/ml (“crude” peptide concentration). The solution was then sonicated in a bath sonicator (Branson, model 2510) for 10 min. The peptide solution at a concentration of 0.5 mg/mL was used to study the differences among the three peptides in self-assembled nanostructures, hydrophobicity and surface activity.

The peptide-ellipticine complexes were prepared by adding 1 mL of the fresh peptide solution into a glass vial containing a thin film of 0.04 mg ellipticine at the bottom, followed by mechanical stirring at 900 rpm for 24 h. 1 mL of pure water, instead of peptide solution, was also added to another vial to make a control sample. The purpose of using a relatively low ellipticine concentration of 0.04 mg/mL in this study was to obtain distinguishable cellular toxicity of the complexes and the control sample. To make a thin film of ellipticine at the bottom of the vials, 100 µL of 0.4 mg/mL ellipticine stock solution in THF was transferred to the vials, and dried with gently blowing of filtered air (0.22 µm pore size filter) for ∼5 min. All the vials and solvents were sterilized and the samples were prepared in a biological safety cabinet to avoid possible contamination, for especially cell culture experiments. For dynamic light scattering (DLS) measurements, the solvents were filtered, and the samples were made in the biosafety cabinet to eliminate potential dust contamination. The complexes were photographed with a digital camera (Cannon PowerShot A95) and characterized with several techniques to obtain complex dimensions and molecular states of the ellipticine in the complexes.

### Determining the maximum suspension concentration of ellipticine

The amount of suspended ellipticine in solution was determined by the ellipticine UV-absorption. The peptide-ellipticine suspension was diluted 20 times in DMSO (resulting in a solvent mixture of 95% DMSO and 5% water by volume) to dissolve ellipticine from the complexes. 80 µL of the solution were then transferred to a quartz microcell (70 µL) with a 1 cm light path and tested on a UV-Vis spectrophotometer (Biochrom Ultraspec 4300 Pro, Cambridge, England). The absorbance at 295 nm was converted to the ellipticine concentration using Beer-Lambert's law: absorbance (Abs) = *εcd*, where *ε* is the molar extinction coefficient, *c* is the molar concentration of ellipticine, and *d* is the optical path length (cm) [37]. The extinction coefficient was obtained as 59000±1100 (R^2^>0.995) from the linear fitting of ellipticine absorption as a function of ellipticine concentration (2–20 µM) prepared in a mixture of 95% DMSO and 5% water. The suspension concentration of ellipticine was averaged from 3 measurements, and compared with the given ellipticine concentration of 0.04 mg/mL. Since not all ellipticine in the thin film at the bottom of the vials could be stabilized and suspended in solution, the comparison of the suspension concentration with the given ellipticine concentration (0.04 mg/mL) would thus provide the maximum percentage of the ellipticine suspension at each formulation condition.

### Atomic Force Microscopy (AFM)

The peptide self-assembled nanostructures were imaged on a PicoScan™ AFM (Molecular Imaging, Phoenix, AZ) in pure water. The samples were prepared with the following procedure: 10 µL of 0.5 mg/mL peptide solution (∼15 min after solution preparation) were put on a freshly cleaved mica substrate, which was fixed on an AFM sample plate; a custom made AFM liquid cell was fastened on top of the mica substrate. The solution was incubated for 10 s to allow the peptide assemblies to adhere to the mica surface. The surface was then washed with pure water 15 times, and 500 µL of pure water were added into the cell prior to AFM imaging. A scanner with a maximum scan area of 6×6 µm^2^ was used to acquire the AFM images. It was operated with a tapping mode using silicon nitride cantilevers with a nominal spring constant of 0.58 N/m (DNP-S, Digital Instruments, Santa Barbara, CA) and a typical tip radius of 10 nm. For the best imaging quality, the tapping frequency was typically set between 16 kHz and 18 kHz and the scan rates controlled between 0.8 and 1 line/s. The experiments were conducted in an environmentally-controlled chamber at room temperature to avoid evaporation of the solution. All AFM images were obtained at a resolution of 256×256 pixels.

### Surface tension measurements

The dynamic surface tension of fresh peptide solutions was measured over a period of 2 h using the Axisymmtratic Drop Shape Analysis-Profile (ADSA-P) technique. The experimental setup and operation of ADSA-P were described in an earlier publication [38] and the references therein.

### Fluorescence spectroscopy

The hydrophobicity of the three peptides and their assemblies was investigated via ANS fluorescence [31], [39]. 10 µM ANS solution was prepared in a 10 mM phosphate buffer at pH 6. The fresh peptide solutions were mixed with the same volume of the ANS solution on a vortex mixer for 10 s. The ANS solution was also mixed with the same volume of pure water as a control sample. 60 µL of the mixed solution were transferred to a quartz microcell and tested on a spectrafluorometer (Photon Technology International, Type QM4-SE, London, Canada) with a continuous xenon lamp as the light source. The sample was excited at 360 nm and the emission spectra were collected from 420 to 670 nm. The excitation and emission slit widths were set at 0.5 mm and 1.25 mm, respectively (0.5 and 1.25 mm corresponds to 2 and 5 nm band path). The spectra were normalized with light scattering of air at 360 nm, to correct the lamp fluctuations.

To study the molecular states of ellipticine in the complexes, 60 µL of the peptide-ellipticine suspensions were transferred to a microcell and tested on the spectrafluorometer. The excitation was set to be 294 nm and the emission was collected from 320 to 650 nm. The excitation and emission slit widths were set at 0.5 mm and 0.25 mm, respectively. The intensities were corrected with an ellipticine standard (2 µM in ethanol, sealed and degassed), to account for lamp fluctuations.

### Dynamic Light Scattering (DLS)

The dimension of the peptide assemblies (0.5 mg/mL) and the complexes from the peptide-ellipticine suspensions was investigated on a Zetasizer Nano ZS (Malvern Instruments, Worcestershire, U.K.) with appropriate viscosity and refractive index settings, and the temperature was maintained at 25°C during the measurement. A quartz microcell (45 µL) with a 3 mm light path was used. The scattered light intensities of the samples at the angle of 173° were collected. The intensity-based size distribution was obtained with the multimodal algorithm CONTIN [40], provided in the software package Dispersion Technology Software 5.0 (Malvern Instruments, Worcestershire, U.K.). Three measurements were performed to generate the intensity-based size distribution plot reported herein.

### Scanning Electron Microscopy (SEM)

A LEO model 1530 field emission SEM (GmbH, Oberkochen, Germany) was employed to study the morphology and dimensions of the peptide-ellipticine complexes. The SEM sample was prepared by depositing 10 µL of the peptide-ellipticine suspensions on a freshly cleaved mica surface. The mica was affixed on an SEM stub using a conductive carbon tape. The sample was placed under a Petridish-cover for 10 min to allow the complexes to adhere onto the mica surface. It was then washed once with a total of 100 µL pure water and air-dried in a desicator overnight. All samples were coated with a 20 nm thick gold layer prior to SEM imaging; the images were acquired using the secondary electron (SE2) mode at 5 kV.

### *In vitro* cell viability studies

Two types of cancer cells, non-small cell lung cancer cell A549 and breast cancer cell MCF-7 (courtesy from Dr. Mingyao Liu at the University of Toronto), were used for *in vitro* cellular toxicity studies on the peptide-ellipticine complexes. The cells were cultured in DMEM containing 10% FBS and 1% p/s at 37°C and with 5% CO_2_. When cells grew to reach ∼95% confluence, they were detached from cell culture flasks with trypsin-EDTA and resuspended in the cell culture media at concentrations of 5×10^4^ and 1×10^5^ cells/mL for A549 and MCF-7 cells, respectively. For each type of cell, 200 µL of the cell suspensions were added into each well of a clear, flat bottom 96-well plate (Costar) and incubated overnight. 50 µL of the treatments (including the complexes and control samples) were then added to the wells each containing 150 µL of fresh culture media. The plates were incubated for 48 h prior to perform the cell viability assay.

MTT assay was used to determine the cell viability after different treatments. 5 mg of solid MTT was dissolved in 3 mL PBS solution, followed by 10 times dilution in the culture medium. All the treatments were taken out before 100 µL of the MTT solution was added to each well of the treated plates. The plates were incubated for 4 h prior to the addition of 100 µL of the solubilization solution (anhydrous isopropanol with 0.1 N HCl and 10% Triton X-100). After overnight incubation, the absorbance at 570 nm was recorded on a microplate reader (BMG FLUOstar OPTIMA) and subtracted by the background signals at 690 nm. The absorption intensities were averaged from 4 replicates for each treatment and normalized to that obtained from the untreated cells (negative control) to generate the cell viability.

## Supporting Information

Figure S1 Mass spectrum of EAK16-II.
(0.50 MB TIF)

Figure S2 Mass spectrum of EAK16-IV.
(0.09 MB TIF)

Figure S3 Mass spectrum of EFK16-II.
(0.53 MB TIF)

Figure S4 HPLC data of EAK16-II. The purity of the peptide is around 73%.
(0.96 MB TIF)

Figure S5 HPLC data of EAK16-IV. The purity of the peptide is around 83%.
(0.24 MB TIF)

## References

[1] Zhang S (2003). Fabrication of novel biomaterials through molecular self-assembly.. Nature Biotech.

[2] Aggeli A, Boden N, Zhang S (1999). Self-assembly of peptides in medicine: two sides of the coin.. Mol Med Today.

[3] Fung SY, Hong Y, Dhadwar SS, Zhao X, Chen P, Nalwa HS (2005). Self-assembly of ionic-complementary peptides and their applications in nanobiotechnology.. Handbook of Nanostructured Biomaterials and Their Applications in Nanobiotechnology.

[4] Zhang S, Holmes T, Lockshin C, Rich A (1993). Spontaneous assembly of a self-complementary oligopeptide to form a stable macroscopic membrane.. Proc Natl Acad Sci USA.

[5] Zhang S, Lockshin C, Cook R, Rich A (1994). Unusually stable β-sheet formation in an ionic self-complementary oligopeptide.. Biopolymers.

[6] Zhang S, Holmes T, DiPersio CM, Hynes RO, Su X (1995). Self-complementary oligopeptide matrices support mammalian cell attachment.. Biomaterials.

[7] Davis ME, Michael Motion JP, Narmoneva DA, Takahashi T, Hakuno D (2005). Injectable self-assembling peptide nanofibers create intramyocardial microenvironments for endothelial cells.. Circulation.

[8] Ellis-Behnke RG, Liang Y-X, You S-W, Tay DKC, Zhang S (2006). Nano neuro knitting: Peptide nanofiber scaffold for brain repair and axon regeneration with functional return of vision.. Proc Natl Acad Sci USA.

[9] Bokhari MA, Akay G, Zhang S, Birch MA (2005). The enhancement of osteoblast growth and differentiation in vitro on a peptide hydrogel−polyHIPE polymer hybrid material.. Biomaterials.

[10] Holmes TC, de Lacalle S, Su X, Liu G, Rich A (2000). Extensive neurite outgrowth and active synapse formation on self-assembling peptide scaffolds.. Proc Natl Acad Sci USA.

[11] Kisiday J, Jin M, Kurz B, Hung H, Semino C (2002). Self-assembling peptide hydrogel fosters chondrocyte extracellular matrix production and cell division: implications for cartilage tissue repair.. Proc Natl Acad Sci USA.

[12] Ellis-Behnke RG, Liang Y-X, Tay DKC, Kau PWF, Schneider GE (2006). Nano hemostat solution: immediate hemostasis at the nanoscale.. Nanomedicine: Nanotech Biol Med.

[13] Schwartz JJ, Zhang S (2000). Peptide-mediated cellular delivery.. Curr Opin Mol Ther.

[14] Keyes-Baig C, Duhamel J, Fung SY, Bezaire J, Chen P (2004). Self-assembling peptide as a potential carrier for hydrophobic compounds.. J Am Chem Soc.

[15] Nagai Y, Unsworth LD, Koutsopoulos S, Zhang S (2006). Slow release of molecules in self-assembling peptide nanofiber scaffold.. J Control Release.

[16] Davis ME, Hsieh PCH, Takahashi T, Song Q, Zhang S (2006). Local myocardial insulin-like growth factor 1 (IGF-1) delivery with biotinlylated peptide nanofibers improves cell therapy for myocardial infarction.. Proc Natl Acad Sci USA.

[17] Hsieh PCH, Davis ME, Gannon J, MacGillivray C, Lee RT (2006). Controlled delivery of PDGF-BB for myocardial protection using injectable self-assembling peptide nanofibers.. J Clin Invest.

[18] Hsieh PCH, MacGillivray C, Gannon J, Cruz FU, Lee RT (2006). Local controlled intramyocardial delivery of platelet-derived growth factor improves postinfarction ventricular function without pulmonary toxicity.. Circulation.

[19] Segers VF, Loffredo F, Tokunou T, Higgins LJ, MacGillivray C (2007). Intramyocardial delivery of protease-resistant stromal cell derived factor-1 by self-assembling peptide nanofibers.. Circulation.

[20] Fung SY, Yang H, Chen P (2007). Formation of colloidal suspension of hydrophobic compounds with an amphiphilic self-assembling peptide.. Colloid Surfaces B: Biointerfaces.

[21] Fung SY, Yang H, Bhola PT, Sadatmousavi P, Muzar E (2008). Self-assembling peptide as potential carrier for hydrophobic anticancer drug delivery: complexation and release.. ACS Nano..

[22] Hong Y, Legge RL, Zhang S, Chen P (2003). Effect of amino acid sequence and pH on nanofiber formation with self-assembling peptides EAK16-II and EAK16-IV.. Biomacromolecules.

[23] Jun S, Hong Y, Imamura H, Ha B-Y, Bechhoefer J (2004). Self-assembly of the ionic peptide EAK16: the effect of charge distributions on self-assembly.. Biophys J.

[24] Caplan MR, Schwartzfarb EM, Zhang S, Kamm RD, Lauffenburger DA (2002). Control of self-assembling oligopeptide matrix formation through systematic variation of amino acid sequence.. Biomaterials.

[25] Gu FX, Karnik R, Wang AZ, Alexis F, Levy-Nissenbaum E (2007). Targeted nanoparticles for cancer therapy.. Nanotoday.

[26] Lundberg P, Langel U (2003). A brief introduction to cell-penetrating peptides.. J Mol Recognit.

[27] Temsamani J, Vidal P (2004). The use of cell-penetrating peptides for drug delivery.. Drug Discov Today.

[28] Eastoe J, Dalton JS (2000). Dynamic surface tension and adsorption mechanisms of surfactants at the air-water interface.. Adv Colloid Interface Sci.

[29] Torrent J, Alvarez-Martinez MT, Harricane M-C, Heitz F, Liautard J-P (2004). High pressure induces scrapie-like prion protein misfolding and amyloid fibril formation.. Biochemistry.

[30] Lindgren M, Sorgierd K, Hammarstrom P (2005). Detection and characterization of aggregates,. prefibrillar amyloidogenic oligomers, and protofibrils using fluorescence spectroscopy.. Biophys J.

[31] Slavik J (1982). Anilinonaphthalene sulfonate as a probe of membrane composition and function.. Biochim Biophys Acta.

[32] Biswas ME, Chatzis I, Ioannidis MA, Chen P (2005). Modeling of adsorption dynamics at air–liquid interfaces using statistical rate theory (SRT).. J Colloid Interface Sci.

[33] Garbett NC, Graves DE (2004). Extending nature's leads: the anticancer agent ellipticine.. Curr Med Chem.

[34] Fung SY, Duhamel J, Chen P (2006). Solvent effect on the photophysical properties of the anticancer agent ellipticine.. J Phys Chem A.

[35] Liu J, Xiao Y, Allen C (2004). Polymer-drug compatibility: a guide to the development of delivery systems for the anticancer agent, ellipticine.. J Pharm Sci.

[36] Yang H, Fung SY, Pritzker MD, Chen P (2007). Surface-assisted assembly of an ionic-complementary peptide: controllable growth of nanofibers.. J Am Chem Soc.

[37] Lakowicz JR (1999). Principles of Fluorescence Spectroscopy..

[38] Yang H, Pritzker M, Fung SY, Sheng Y, Wang W (2006). Anion effect on the nanostructure of a metal ion binding self-assembling peptide.. Langmuir.

[39] Cardamone M, Puri NK (1992). Spectrofluorimetric assessment of the surface hydrophobicity of proteins.. Biochem J.

[40] Provencher SW (1982). A constrained regulation method for inverting data represented by linear algebraic or integral equations.. Comput Phys Commun.

