# Supplementary figures and images for: Sequence Effect of Self-Assembling Peptides on the Complexation and In Vitro Delivery of the Hydrophobic Anticancer Drug Ellipticine
Source: PLoS One. 2008 Apr 9;3(4):e1956. doi: 10.1371/journal.pone.0001956 (PMC2276859; doi:10.1371/journal.pone.0001956)

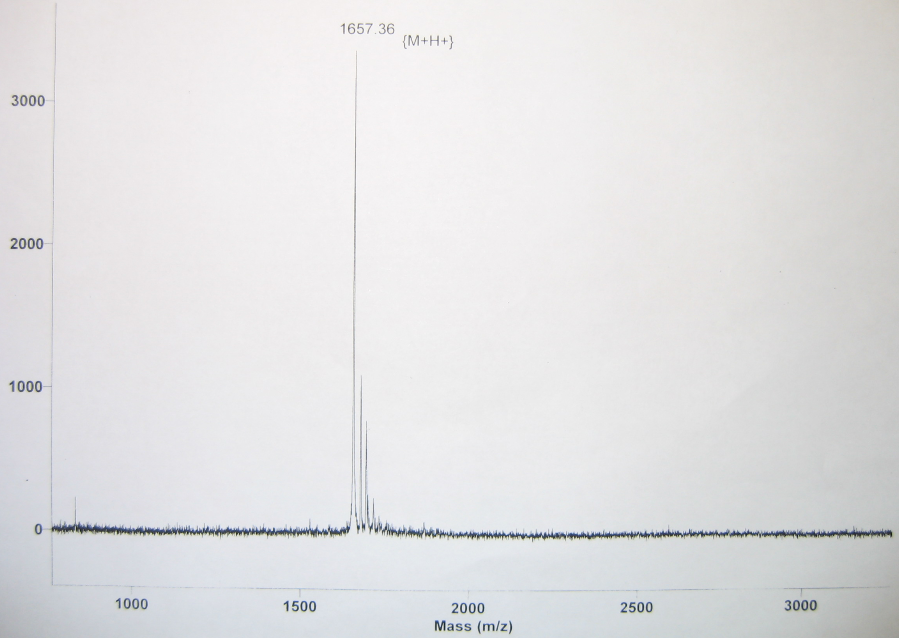

Supplement: Figure S1 — Mass spectrum of EAK16-II. (0.50 MB TIF) [file pone.0001956.s001.tif]

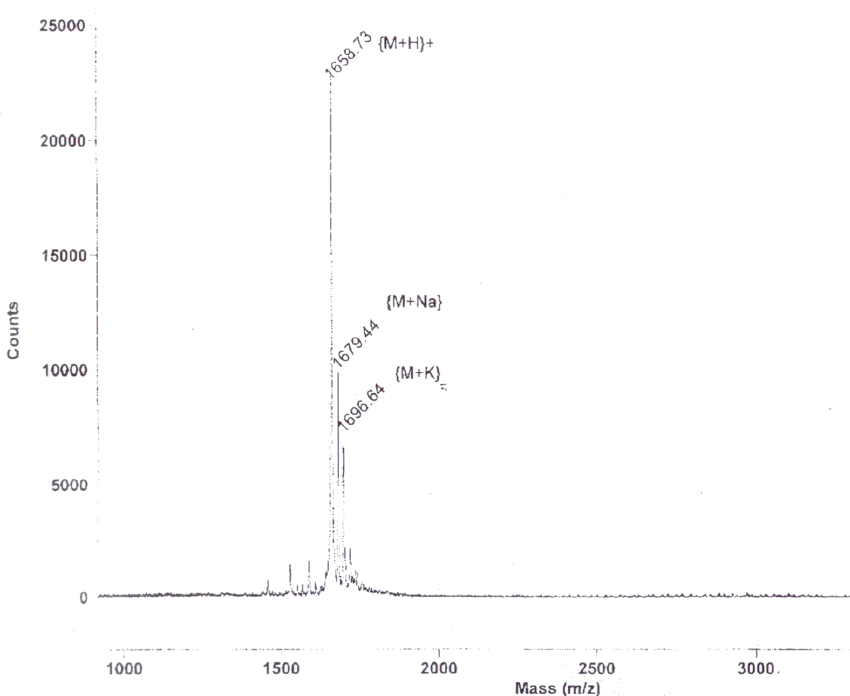

Supplement: Figure S2 — Mass spectrum of EAK16-IV. (0.09 MB TIF) [file pone.0001956.s002.tif]

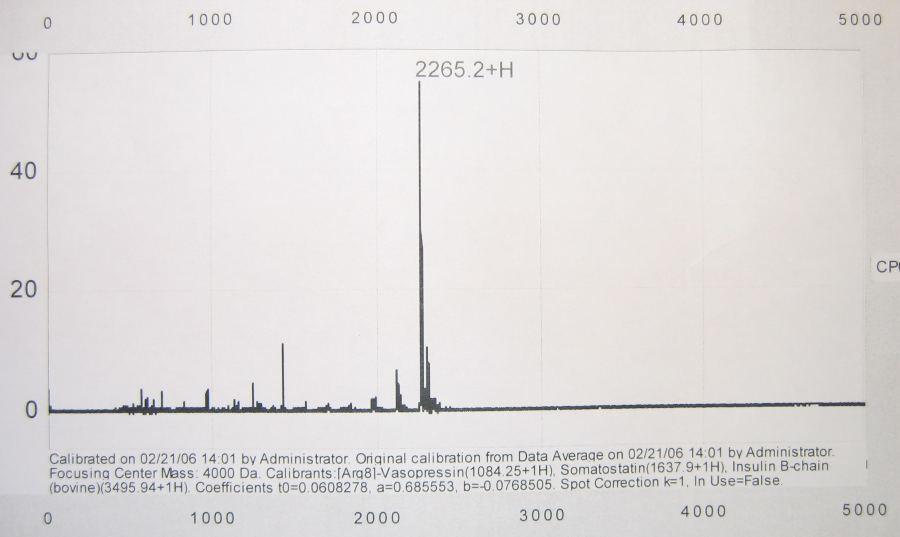

Supplement: Figure S3 — Mass spectrum of EFK16-II. (0.53 MB TIF) [file pone.0001956.s003.tif]

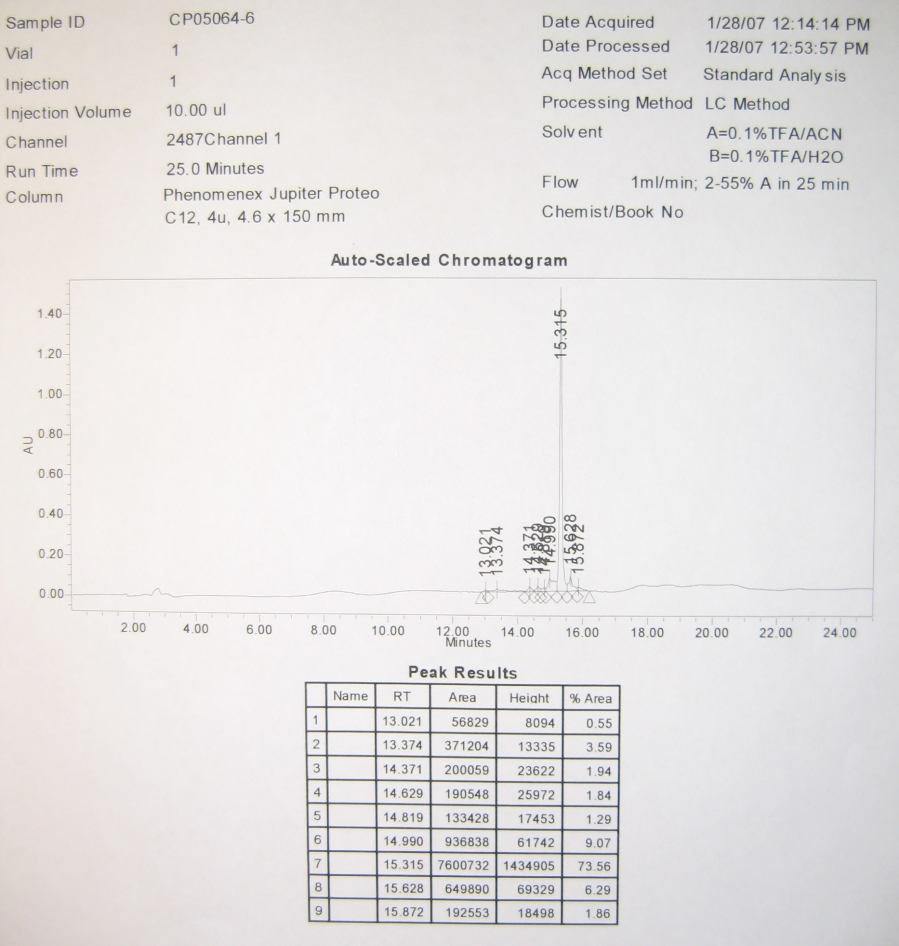

Supplement: Figure S4 — HPLC data of EAK16-II. The purity of the peptide is around 73%. (0.96 MB TIF) [file pone.0001956.s004.tif]

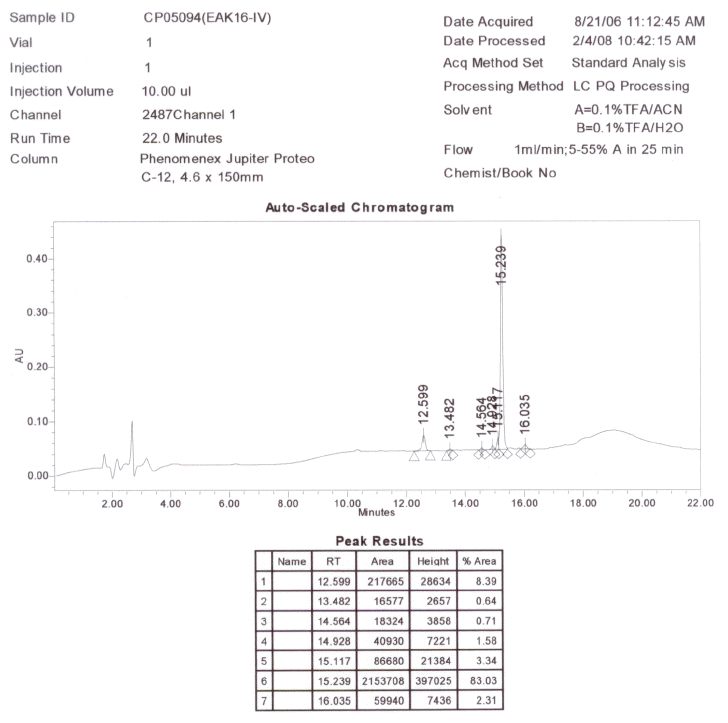

Supplement: Figure S5 — HPLC data of EAK16-IV. The purity of the peptide is around 83%. (0.24 MB TIF) [file pone.0001956.s005.tif]
